# Raman Spectroscopy of Two-Dimensional Bi_2_Te*_x_*Se_3 − *x*_ Platelets Produced by Solvothermal Method

**DOI:** 10.3390/ma8085007

**Published:** 2015-08-05

**Authors:** Jian Yuan, Meng Zhao, Wengzhi Yu, Yao Lu, Caiyun Chen, Meng Xu, Shaojuan Li, Kian Ping Loh, Qiaoliang Bao

**Affiliations:** 1 Institute of Functional Nano and Soft Materials (FUNSOM), Jiangsu Key Laboratory for Carbon-Based Functional Materials and Devices, and Collaborative Innovation Center of Suzhou Nano Science and Technology, Soochow University, Suzhou 215123, China; E-Mails: 20124214040@suda.edu.cn (J.Y.); 20144214003@stu.suda.edu.cn (W.Y.); 20134214095@stu.suda.edu.cn (Y.L.); 20124214017@suda.edu.cn (C.C.); xujane1225@gmail.com (M.X.); sjli@suda.edu.cn (S.L.); 2 Department of Chemistry, and Graphene Research Centre, National University of Singapore, 3 Science Drive 3, Singapore 117543, Singapore; E-Mails: zhaomeng@nus.edu.sg (M.Z.); chmlohkp@nus.edu.sg (K.P.L.); 3 Department of Materials Science and Engineering, Monash University, Clayton, VIC 3800, Australia

**Keywords:** bismuth chalcogenide, solvothermal, stoichiometric ratio, Raman spectroscopy, infrared active

## Abstract

In this paper, we report a facile solvothermal method to produce both binary and ternary compounds of bismuth chalcogenides in the form of Bi_2_Te*_x_*Se_3 − *x*_. The crystal morphology in terms of geometry and thickness as well as the stoichiometric ratio can be well controlled, which offers the opportunities to systematically investigate the relationship between microstructure and phonon scattering by Raman spectroscopy. Raman spectra of four compounds, *i.e.*, Bi_2_Se_3_, Bi_2_Se_2_Te, Bi_2_SeTe_2_ and Bi_2_Te_3_, were collected at four different excitation photon energies (2.54, 2.41, 1.96, and 1.58 eV). It is found that the vibrational modes are shifted to higher frequency with more Se incorporation towards the replacement of Te. The dependence of Raman vibrational modes on excitation photon energy was investigated. As the excitation photon energy increases, three Raman vibrational modes (A_1g_^1^, E_g_^2^ and A_1g_^2^) of the as-produced compounds move to low frequency. Three Infrared-active (IR-active) modes were observed in thin topological insulators (TIs) crystals.

## 1. Introduction

Topological insulators (TIs) such as bismuth telluride, antimony telluride and other binary/ternary group V–VI compounds, are emerging electronic materials that have a bulk band gap similar to conventional insulator but possessing protected conducting states on their edge or surface [1,2,3]. Owing to the emerging theoretical prediction of transport without dissipation in two-dimensional (2D) TIs, diverse experimental works about the quantum-spin Hall effects have been reported [4,5,6]. The exotic physical properties of 2D TIs have stimulated extensive range of applications for electronic [7], optoelectronic [8], and thermoelectric devices [9]. The fast progress in the area demands large scale production of high-quality 2D TI crystals with desired structure and properties. However, it is still challenging to control the crystal morphology perfectly in terms of geometry and thickness as well as the stoichiometric ratio [3,10]. Furthermore, the light-matter interactions in 2D TIs with respect to phonon scattering and the dependence of these interactions on the microstructure are not well understood [11].

In this work, we present a facile solvothermal method to synthesize Bi_2_Te*_x_*Se_3 − *x*_ platelets in large scale and with high yield [12,13,14,15,16]. The use of ethylene glycol (EG) as both reducing agent and solvent makes the synthesis process simple and friendly [17]. The systematic material characterizations on four products (Bi_2_Se_3_, Bi_2_Se_2_Te, Bi_2_SeTe_2_ and Bi_2_Te_3_) indicate the successful growth of TI platelets with controlled morphology and high quality. The systematic Raman spectroscopy characterizations reveal that the characteristic peaks are dependent on the photon energy as well as the elemental composition and new vibration modes can be observed with lower photon energy.

## 2. Experimental

### 2.1. Material Synthesis

Bismuth nitrate (Bi(NO_3_)_3_), bismuth oxide (Bi_2_O_3_), seleniumoxide (SeO_2_) and tellurium oxide (TeO_2_) were purchased from Alfa Aesar. Polyvinylpyrrolidone (PVP, K30) were purchased from TCI, Sodium hydroxide (NaOH) and ethylene glycol (EG) was acquired from Shanghai Chemical Reagent Co. (Shanghai, China) All the chemicals used for the synthesis of Bi_2_Te*_x_*Se_3 − *x*_ sheets in the present work are of analytical grade without further purification.

For the synthesis of Bi_2_Te_3_, 0.5 mmol Bi(NO_3_)_3_, 0.75 mmol TeO_2_, 2 mL NaOH (8 mol/L) and 0.4 g PVP were mixed and dissolved in 18 mL EG, followed by stirring for 1 h and the mixture was then transferred into a stainless steel autoclave with Teflon lining up to 40%–60% of the capacity. The autoclave was heated at 200 °C in an oven for 24 h and then cooled down to room temperature. The residual gray powders were washed with deionized water and ethanol and finally dried at 60 °C in a vacuum chamber overnight. The other three compounds (Bi_2_Te_2_Se, Bi_2_TeSe_2_ and Bi_2_Se_3_) were also synthesized by the similar procedure as described above. The differences from the previous growth are the change in their reaction temperature as 220 °C and the concentrations of NaOH are maintained at 4, 1 and 0.5 mol/L, respectively.

### 2.2. Material Characterizations

The phase structure of the final products was investigated by X-ray diffractometer (XRD, PANAlytical, EMPYREAN, Almelo, The Netherlands) using Cu Kα radiation (λ = 1.541 Å). Scanning electron microscopy (SEM, FEI Quanta 200 FEG, FEI, Hillsboro, OR, USA) was employed to study the morphology of the products. The microstructure was examined with the aid of high-resolution transmission electron microscopy (HRTEM, FEI Tecnai G2 F20 STWIN, FEI). Raman spectra of nanostructured Bi_2_Te*_x_*Se_3 − *x*_ samples were recorded using confocal Raman spectrometer (Horiba Jobin Yvon, Labram HR 800, Horiba Jobin Yvon, Paris, France) with four different excitation photon energy (2.54 eV (488 nm), 2.41 eV (514 nm), 1.96 eV (633 nm), and 1.58 eV (785 nm)) at room temperature. The laser power is fixed to be ~4 × 10^5^ W/cm^−2^ at different wavelengths. The measured spectral range is 10–200 cm^−1^ and the spectral resolution is 0.5 cm^−1^ using the 1800 groove/mm grating. The topography and thickness of the as-produced samples were determined by atomic force microscope (AFM, Bruker, Veeco Multimode V, Bruker, Santa Barbara, CA, USA).

## 3. Results and Discussion

The material growth parameters are very critical in the synthesis of both binary and ternary A_2_^V^B_3_^VI^-type hexagonal platelets via solvothermal method. It is generally believed that the reaction time, reaction temperature, the concentration of alkalis and the surfactants (PVP) are key factors that affect the crystallization of Bi_2_Te*_x_*Se_3 − *x*_ [14]. The formation of Bi_2_Te*_x_*Se_3 − *x*_ platelets includes two major steps: (i) reduction of Bi_2_O_3_ and TeO_2_/SeO_2_ by EG; and (ii) oriented attachment of the Bi-Te/Bi-Se alloy particles into crystalline platelets with hexagonal shape [13]. We have found that NaOH plays a vital role in the reduction process regarding step (i) and PVP serve as a template for the crystal growth in step (ii), which is discussed in detail elsewhere [18].

**Figure 1:**
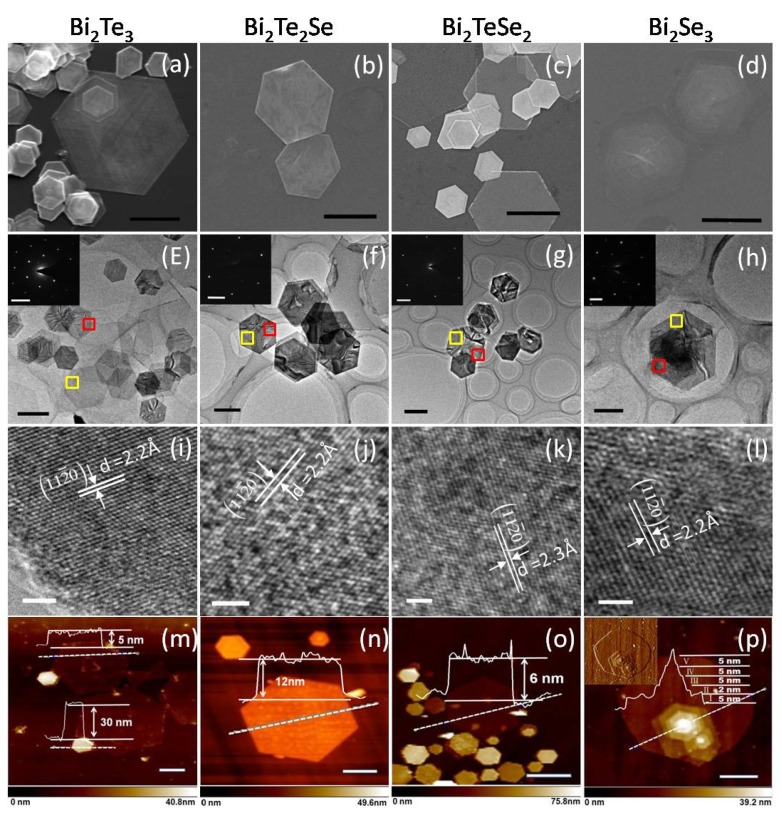
Characterizations of Bi_2_Te_3_, Bi_2_Te_2_Se, Bi_2_TeSe_2_ and Bi_2_Se_3_ platelets. (**a**–**d**) Scanning electron microscopy (SEM) and (**e**–**h**) transmission electron microscopy (TEM) images of these four compounds. The insets of (**e**–**h**) are selected area electron diffraction (SAED) patterns taken at the red square area in the TEM image. Scale bars in (**a**–**d**) are 1 μm. Scale bars in (**e**–**h**) are 500 nm. (**i**–**l**) High-resolution transmission electron microscopy (HRTEM) images taken at the yellow square area in TEM images (**e**–**h**). Scale bars: 2 nm. (**m**–**p**) Typical atomic force microscope (AFM) images and height profiles of Bi_2_Te_3_, Bi_2_Te_2_Se, Bi_2_TeSe_2_ and Bi_2_Se_3_ platelets. The height profiles floating on the images were taken at the dashed lines. The inset in (**p**) shows the phase image. Scale bars: 1 μm.

Figure 1 shows the materials characterization data of the as-produced four compounds, *i.e.*, Bi_2_Se_3_, Bi_2_Se_2_Te, Bi_2_SeTe_2_ and Bi_2_Te_3_. Figure 1a depicts the typical SEM image of Bi_2_Te_3_ platelets. It is clearly seen that the high-yield hexagonal nanosheets were obtained with the size ranging from 400 nm to 5 μm. The regular shape and sharp edges indicate excellent crystallinity. Figure 1e shows the typical TEM image of Bi_2_Te_3_ platelets, in which the relative uniform contrast demonstrates a small difference in thickness. The selected area electron diffraction (SAED) pattern shown in the inset of Figure 1e can be indexed to the [001] zone axis of rhombohedral Bi_2_Te_3_, indicating that the nanosheet is single crystal with a preferential (001) orientation. As the top-bottom facets are indexed to be {0001} while the six edge facets are {11$\bar{2}$0}, the growth of the hexagon platelet is determined to be along 〈11$\bar{2}$0〉 direction, which is consistent with the previous report. The HRTEM image in Figure 1i clearly shows that the lattice fringes are structurally uniform with a spacing of 2.2 Å and is in good agreement with that of the (110) planes of rhombohedral phase. The representative AFM topography is shown in Figure 1m where the Bi_2_Te_3_ platelet has a flat surface with thickness of 12 nm.

Figure 1b,c shows the SEM morphologies of ternary V-VI based alloys Bi_2_Te_2_Se and Bi_2_TeSe_2_, in which hexagonal platelets are observed. The microstructure of these two compounds shown in Figure 1f,g confirms the nature of single crystal with rhombohedral symmetry. The size of the platelets is in the average of about 500 nm to a few micrometers. The HRTEM images in Figure 1j,k clearly reveal the lattice of {11$\bar{2}$0} facet with spacing of 2.2 Å and 2.3 Å, respectively. It was examined that the ternary platelets are well-crystallized with less atomic defects, which are similar to those of Bi_2_Te_3_ platelets. The AFM images shown in Figure 1n,o indicate that the platelets have a thickness in the range of 6 to 25 nm.

Figure 1d,h show representative SEM and TEM images of Bi_2_Se_3_ platelets. It is interesting to see that these hexagonal crystals are stacking together, indicating that these platelets nucleate from the same seed. The SAED result taken from bilayer region (red square in the inset of Figure 1h) presents only one set of hexagonal pattern, suggesting that these two layers are of the same crystal orientation. The SAED pattern can be indexed to be [001] zone axis of the rhombohedral Bi_2_Se_3_. The multilayer stacking can be further confirmed by AFM topography and phase images, as shown in Figure 1p and its inset. The height profile (Figure 1p) displays obvious plateaus with step height of about 5 nm. The HRTEM image in Figure 1l clearly presents the lattice fringes, which are structurally uniform with spacing of 2.2 Å, and is in good agreement with the previous report [19].

**Figure 2:**
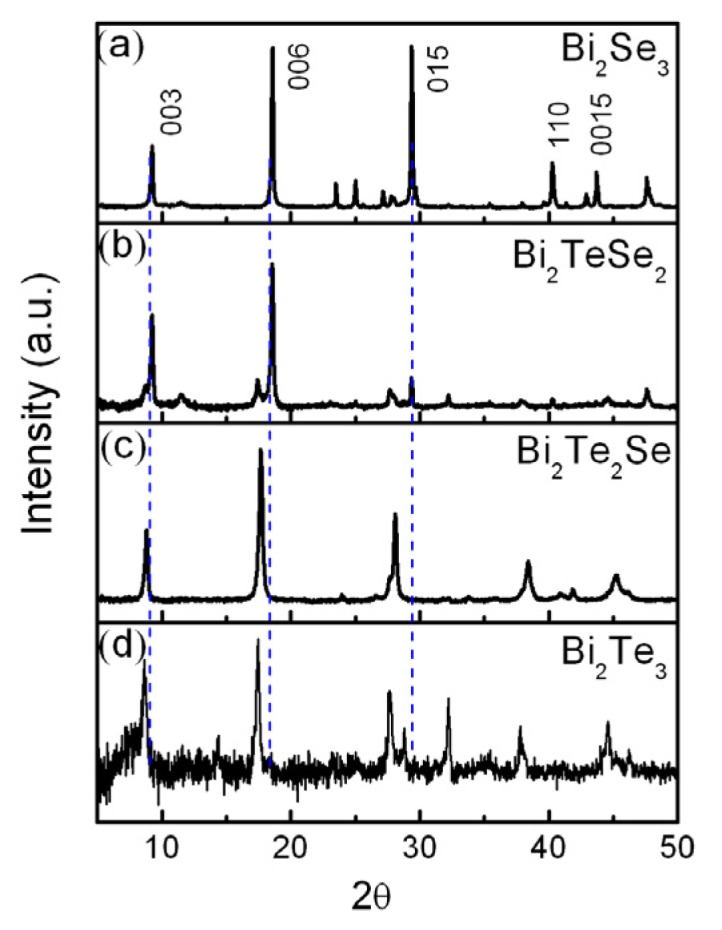
X-ray diffractometer (XRD) patterns of the as-grown four compounds (Bi_2_Te_3_, Bi_2_Te_2_Se, Bi_2_TeSe_2_ and Bi_2_Se_3_).

The crystal structure and phase of the as-prepared samples were verified by XRD, as shown in Figure 2. Three strongest peaks in each spectrum can be fully indexed to the reflections of three facets (*i.e.*, (003), (006) and (0015)) in rhombohedral phase. On the basis of XRD pattern, Bi_2_Se_3_, possessing a preferred growth direction of (006), is assigned to have rhombohedral phase with lattice constants *a* = 4.140 Å, *c* = 28.636 Å (refer to JCPDS Card Number 82-0358) [13], space group: *R*$\bar{3}$*m* (${\text{D}}_{3\text{d}}^{5}$). The lattice constants of Bi_2_TeSe_2_ is found to be relatively close to that of Bi_2_Se_3_, *i.e.*, *a* = 4.218 Å, *c* = 29.240 Å. By contrast, the lattice parameters of the other two compounds (Bi_2_SeTe_2_ and Bi_2_Te_3_) are slightly larger, *a* = 4.303 Å, *c* = 30.010 Å for Bi_2_SeTe_2_ and *a* = 4.390 Å, *c* = 30.460 Å for Bi_2_Te_3_. This suggests that the unit cells of Bi_2_SeTe_2_ and Bi_2_Te_3_ are expanded along the in-plane and out-of-plane directions (*i.e.*, along both *a* and *c* directions) due to the bonding with larger atom Te.

**Figure 3:**
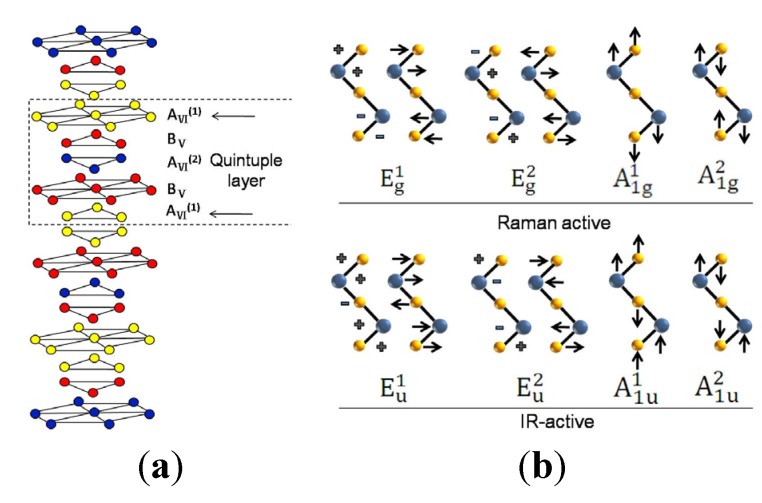
(**a**) Schematic diagram of Bi_2_Te*_x_*Se_3 − *x*_ crystal structure. (**b**) Schematic diagram of Raman active and Infrared-active (IR-active) vibration modes.

In order to determine the vibrational modes, it is important to investigate the atomic structure and chemical bonds of this group of compounds. According to XRD results, all four compounds are assigned to be layered rhombohedral crystal with space group *R*$\bar{3}$*m* (${\text{D}}_{3\text{d}}^{5}$), which is schematically shown in Figure 3a. These materials consist of anisotropic layers in which five mono-atomic planes are covalently bonded to form a quintuple layer. These quintuple layers are weakly bound to each other by the van der Waals forces and can be described as following: –A_VI_^(1)^–B_V_–A_VI_^(2)^–B_V_–A_VI_^(1)^–, where A_VI_ refers to either Te or Se, and B_V_ stands for Bi. The superscripts on the A atoms designate the different positions within the five-fold layers and the five-layer stacks are centro-symmetrical with respect to A_VI_^(2)^, which plays the role of an inversion center [20]. As the primitive unit cell contains five atoms according to the chemical formula, it means that there are 15 lattice vibrational modes. Consequently, there are 15 lattice dynamical modes locate at the Brillouin zone center (*q* = 0), including three acoustic modes and 12 optical modes. Therefore, group theory allows Γ_bulk_-center modes, which decompose in the irreducible representations and can be described as Γ_bulk_ = 2A_1g_ + 3A_2u_ + 2E_g_ + 3E_u_ [21,22]. These phonon modes are exclusively either Raman or infrared (IR) active [23,24] due to the inversion crystal symmetry. Specifically, the A_1g_ and E_g_ modes are Raman active, while the A_2u_ and E_u_ modes of non-zero frequency are IR-active, as illustrated in Figure 3b.

As the A_1g_ mode is attributed to the symmetric out-of-plane stretching of A_VI_-B_V_ atoms vibrating in opposite directions, a short displacement induces higher phonon frequency. Whereas E_g_ mode is caused by symmetric in-plane bending and shearing the upper two layers of A_VI_-B_V_ atoms vibrating in the same direction, which make a greater atomic displacement by producing lower phonon frequency. Since the A_g_ modes are vibrating out of phase, the bonding of each neighboring atom is stronger than that of the E_g_ modes. As a result, the atomic vibration displacement of A_g_ modes is restricted and the atomic vibrational frequency of A_g_ modes is stronger than that of E_g_ modes, which is experimentally verified here in after.

**Figure 4:**
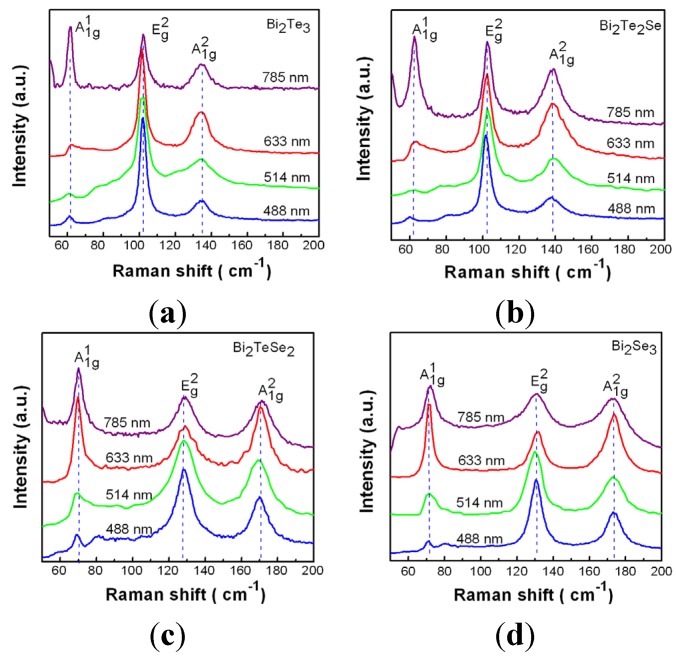
Raman spectra of (**a**) Bi_2_Te_3_, (**b**) Bi_2_Te_2_Se, (**c**) Bi_2_TeSe_2_ and (**d**) Bi_2_Se_3_ measured at four different excitation wavelengths (488 nm (2.54 eV), 514 nm (2.41 eV), 633 nm (1.96 eV), and 785 nm (1.58 eV)).

Figure 4 depicts the Raman spectra of all the four samples measured with four different excitation photon energies (2.54, 2.41, 1.96, and 1.58 eV). We can observe three main peaks in the Raman spectra of these four compounds corresponding to A_1g_^1^, E_g_^2^ and A_1g_^2^, respectively. It is found that stronger resonant peaks especially A_1g_^1^ can be observed with lower excitation photon energy, which may be correlated to the resonant condition and larger skin depth at longer wavelengths. For the Te rich compounds (Bi_2_SeTe_2_ and Bi_2_Te_3_), the stretching modes of A_1g_^1^ and A_1g_^2^ appear at 62 cm^−1^ and 137 cm^−1^, respectively. The A_1g_^2^ mode is significantly red-shift compared with that measured at low temperature (*i.e*., 8 K). [25] The bending mode of E_g_^2^ is found at 102 cm^−1^. For the Se rich compounds (Bi_2_TeSe_2_ and Bi_2_Se_3_), all the three Raman peaks are shifted to higher frequency, *i.e.*, the stretching modes of A_1g_^1^ and A_1g_^2^ appearing at ~72 cm^−1^ and ~172 cm^−1^, respectively, along with the bending mode of E_g_^2^ at ~130 cm^−1^.

**Figure 5:**
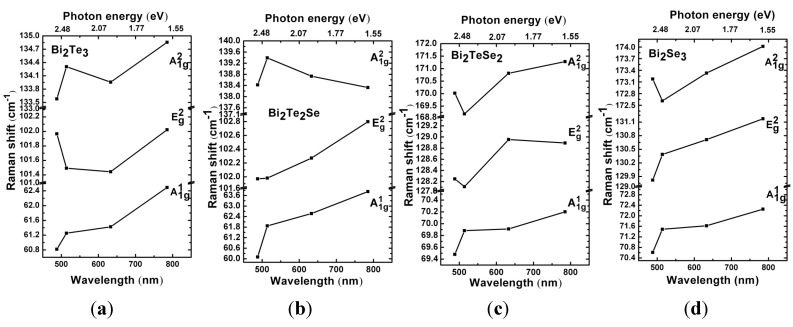
Raman shift of (**a**) Bi_2_Te_3_, (**b**) Bi_2_Te_2_Se, (**c**) Bi_2_TeSe_2_ and (**d**) Bi_2_Se_3_ under different excitation wavelengths (488, 514, 633 and 785 nm).

We further investigate the dependence of frequency of vibrational modes on the photon energy for all four compounds, as shown in Figure 5. The peak positions show weak dependence on the photon energy in terms of small frequency shift, which may be due to the fact that our samples have more than five quintuple layers. However, we can still observe the general trend that these three Raman vibrational modes (especially A_1g_^2^ and E_g_^2^) of the as-produced four compounds shift to higher frequencies while the excitation photon energy varies from 2.54 to 1.58 eV. The frequency dependence observed here is correlated to non-uniform phonon dispersion of these modes throughout the Brillouin zones [26,27]. For example, the A^1^_g_ mode corresponds to the longitudinal phonon vibration, which leads to a resonance on the Γ–Z direction with the increasing of excitation photon energy. As revealed by the phonon dispersion diagrams, [23,26] A^1^_g_ modes of Bi_2_Te*_x_*Se_3 − *x*_ become soft along the Γ–Z direction, resulting in the shift towards lower frequency at Γ point. This is qualitatively in agreement with our experimental observations. Nevertheless, the heating effect induced by the laser with lower photon energy could be another innegligible factor for the observed Raman peak shift.

**Figure 6:**
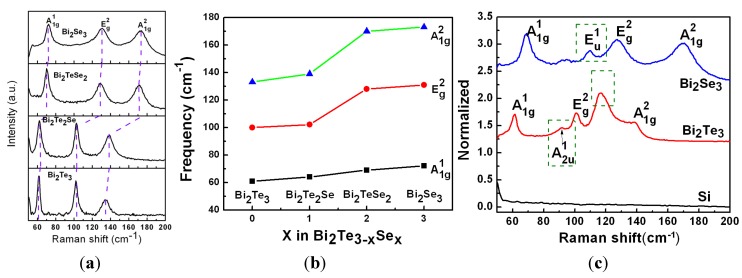
(**a**) Raman spectra of four compounds excited at 785 nm. (**b**) The dependence of Raman peak position on stoichiometric ratio. (**c**) Observation of IR-active vibrational modes in Raman spectra excited at 785 nm. The dashed boxes indicate the peaks of those IR-active modes.

In order to further understand the effect of stoichiometric ratio on vibrational modes, we re-plot the Raman spectra excited at 1.58 eV in Figure 6a,b. The frequency shift of all the Raman modes in the Bi_2_Te*_x_*Se_3 − *x*_ system is observed for *x* ∈ (0, 1, 2, 3), corresponding to the substitution of the Te atoms by Se. It is found that the vibrational modes are shifted to higher frequency with more Se incorporation towards the replacement of Te. This is because of the smaller size of Se atoms with stronger electro-negativity, which leads to the shortening of the chemical bonds between Se and Bi. This observation agrees with the XRD results above-mentioned, which suggest that Se rich compounds have smaller unit cell. It is interesting to observe large peak shift for E_g_^2^ and A_1g_^2^ modes (>25 cm^−1^) while the chemical composition is changed from Bi_2_Te_2_Se to Bi_2_TeSe_2_. In comparison, the position of A_1g_^1^ mode does not have significant shift for the reason that the out-of-plane stretching is not so sensitive to the change of chemical bonds.

Interestingly, we observed two IR-active modes in some TI crystals, *i.e.*, E_u_ mode at 110 cm^−1^ for Bi_2_Se_3_ and A^1^_2u_ at 92 cm^−1^ for Bi_2_Te_3_, as indicated in Figure 6c [27,28,29]. It is assumed that the appearance of these modes is caused by the size effect in the nanoplatelets. In case of Bi_2_Te_3_, the new peak are identified as A_2u_ mode composed of longitudinal optical (LO) phonons at the Brillouin zone boundary (Z point). In principle, the A_2u_ mode is not Raman-active mode but IR-active mode in bulk Bi_2_Te_3_ crystals. However, this IR-active mode is observable in case of symmetry breaking in which infinite crystal periodicity is absent. For example, the A^1^_2u_ mode at 92 cm^−1^ can be explained by the surface phonon mode, which is only observed in nano-sized materials [30]. In case of Bi_2_Se_3_, E_u_ mode at 110 cm^−1^ was experimentally observed for the first time. It has been theoretically predicted that E_u_ mode is correlated to the in-plane vibration, which is only observable in very thin Bi_2_Se_3_ sheet. A prominent vibrational mode at 115 cm^−1^ is also observed in Bi_2_Te_3_, as shown in Figure 6c. According to previous report on bismuth chalcogenide nanoplates [31,32], this new peak of Bi_2_Te_3_ are likely originated from Te impurities because laser can cause Te-containing compound to decompose and lead to strong Te Raman features.

## 4. Conclusions

In summary, we have demonstrated a practical hydrothermal approach to produce Bi_2_Te*_x_*Se_3 − *x*_ platelets in a large scale. Material characterizations reveal that these platelets are single crystalline with thickness of 5 nm to 50 nm and size of 400 nm to 5 μm. A systematic Raman spectroscopy study was performed at four visible excitation wavelengths for all four compounds. It is found that the vibrational modes are shifted to higher frequency with more Se in corporation towards the replacement of Te. The characteristic Raman vibrational modes show a weak dependence on laser excitation energy. We observed two IR-active modes in TI crystals, *i.e.*, E_u_ mode at 110 cm^−1^ for Bi_2_Se_3_, A^1^_2u_ at 92 cm^−1^ for Bi_2_Te_3_, and the appearance of these modes is mainly due to quantum size effects. The main findings in this work will be beneficial to future electronic, photonic and spintronic applications.
